# From Connectivity to Controllability: Unraveling the Brain Biomarkers of Major Depressive Disorder

**DOI:** 10.3390/brainsci14050509

**Published:** 2024-05-17

**Authors:** Chunyu Pan, Ying Ma, Lifei Wang, Yan Zhang, Fei Wang, Xizhe Zhang

**Affiliations:** 1 School of Computer Science and Engineering, Northeastern University, Shenyang 110169, China; chunyupan@foxmail.com; 2 School of Biomedical Engineering and Informatics, Nanjing Medical University, Nanjing 210033, China; gretchen.y.ma@gmail.com (Y.M.); zhangyan2023@stu.njmu.edu.cn (Y.Z.); 3 Early Intervention Unit, Department of Psychiatry, The Affiliated Brain Hospital of Nanjing Medical University, Nanjing 210024, China; lifeiwang@stu.njmu.edu.cn (L.W.); fei.wang@yale.edu (F.W.); 4 Functional Brain Imaging Institute of Nanjing Medical University, Nanjing 210024, China; 5 Department of Mental Health, School of Public Health, Nanjing Medical University, Nanjing 211166, China

**Keywords:** brain network, network controllability, major depressive disorder, fMRI biomarkers

## Abstract

Major Depressive Disorder (MDD) is a significant neurological condition associated with aberrations in brain functional networks. Traditional studies have predominantly analyzed these from a network topology perspective. However, given the brain’s dynamic and complex nature, exploring its mechanisms from a network control standpoint provides a fresh and insightful framework. This research investigates the integration of network controllability and machine learning to pinpoint essential biomarkers for MDD using functional magnetic resonance imaging (fMRI) data. By employing network controllability methods, we identify crucial brain regions that are instrumental in facilitating transitions between brain states. These regions demonstrate the brain’s ability to navigate various functional states, emphasizing the utility of network controllability metrics as potential biomarkers. Furthermore, these metrics elucidate the complex dynamics of MDD and support the development of precision medicine strategies that incorporate machine learning to improve the precision of diagnostics and the efficacy of treatments. This study underscores the value of merging machine learning with network neuroscience to craft personalized interventions that align with the unique pathological profiles of individuals, ultimately enhancing the management and treatment of MDD.

## 1. Introduction

Major Depressive Disorder (MDD) is a prevalent, recurrent psychiatric condition that stands as one of the most significant mental health challenges worldwide. It is characterized by persistent sadness and a marked decrease in interest in activities that were previously rewarding [1]. According to the DSM-V, MDD is diagnosed based on the presence of one or more major depressive episodes (MDEs) and the absence of any manic or hypomanic episodes, with symptoms including depressed mood or anhedonia [2]. MDD affects approximately 322 million people globally, representing 4.4% of the population, and has seen an 18.4% increase from 2005 to 2015 [3]. In China, the prevalence reaches 3.4% [4]. The disorder’s extensive morbidity significantly affects both personal lives and economic structures [5,6], leading the WHO to project it as the primary global disease burden by 2030 [7]. MDD’s prevalence is matched by its heterogeneity; individuals with MDD display a broad spectrum of symptoms, severity levels, treatment outcomes, and pathophysiological bases [3,8]. This diversity not only complicates the diagnostic and therapeutic processes but also underscores the imperative for sophisticated analytical tools to dissect its complex nature [9].

Recent breakthroughs in functional magnetic resonance imaging (fMRI) have significantly advanced our understanding of the brain’s intricate networks, shedding light on the brain underpinnings of MDD [10,11]. These studies reveal marked changes in brain network connectivity among MDD sufferers, underscoring the disorder’s influence on the brain’s operational framework [12,13]. The integration of fMRI with cutting-edge analytical methodologies offers a promising avenue for demystifying the sophisticated network dysfunctions characteristic of MDD, potentially steering the development of more tailored therapeutic strategies [14]. Resting-state fMRI is favored over task-based data primarily because it captures the brain’s intrinsic functional connectivity without the confounding effects of task performance, which can vary significantly among subjects, especially in clinical groups. This method ensures that connectivity measurements are not influenced by an individual’s ability to perform specific tasks. This is particularly relevant in studies involving depressed individuals, who may experience variations in task performance due to decreased concentration, motivation, or cognitive impairments [10].

Resting-state fMRI offers critical insights into the brain’s intrinsic functional networks, particularly the default-mode network (DMN), which plays a significant role in the pathophysiology of depression. Research indicates that altered connectivity within the DMN, which is active during rest and involved in internal processes such as self-reflection, memory retrieval, and future planning, is associated with depression [10,13]. The hyperconnectivity within the DMN, particularly to frontal brain regions, correlates with rumination, a prevalent symptom of depression [15,16]. Additionally, studies have shown that MDD features reduced connectivity between the DMN and the executive control network, vital for mood and affect regulation, highlighting further aspects of network dysfunction in depression [12,14]. Moreover, distinct connectivity patterns in the DMN of MDD patients, as compared to healthy controls, suggest its potential as a biomarker for diagnosing depression [13,17].

The concept of network controllability, adapted from network science and control theory, presents an innovative perspective on the brain’s ability to navigate between various states. This innovative perspective, rooted in the principles of systems theory, posits that the brain’s capacity to transition between various brain states is pivotal for maintaining cognitive and emotional equilibrium [18]. The fundamental goal of network controllability is to drive the network from any initial state to a desired target state via specific input nodes [19]. Currently, network control theory has been applied in various biological networks, such as protein interaction networks [20], virus–protein networks [21], cancer regulatory networks [22], and brain networks [18]. In various network controllability methods, average controllability is considered a significant approach in the context of mental disorders. It measures the ability of a network’s nodes to drive the system into diverse states, providing crucial insights into the neural mechanisms underlying psychiatric conditions [18,23,24,25].

The exploration of network controllability within the human brain presents a transformative approach to understanding and addressing MDD. In the context of brain networks, this concept explores how specific regions or nodes within the brain’s complex network can influence overall network dynamics, thereby facilitating or hindering the transition between different brain states. Leveraging the controllability framework, recent research endeavors have sought to identify critical brain regions that exert a significant influence over the brain’s state dynamics. By quantifying the influence of control points within the brain’s network on its dynamic behavior or how easily a node can be driven to desired states using external inputs [18], researchers are poised to pinpoint pivotal nodes whose adjustment could either reinstate normative function or augment the brain’s receptivity to interventions [26]. These regions are considered essential for modulating neural activity patterns and, by extension, for orchestrating the brain’s shift from disease states to healthier modes of functioning. In the context of MDD, this framework is crucial in understanding the brain’s functional architecture and its ability to adapt or respond to changes, which is particularly relevant in the study of neuropsychiatric disorders like MDD, providing insights into the disorder’s clinical diversity and resistance to conventional treatments [18,27]. Disturbances in these transitional capabilities, particularly within specific neural circuits, are believed to underlie the disorder’s symptomatic manifestations and its resistance to conventional treatments [28,29]. This approach not only promises to deepen our understanding of MDD’s brain underpinnings but also aids in identifying the differential brain regions between individuals with MDD and healthy controls. It heralds the advent of personalized medicine in psychiatry, where interventions are customized to target the distinct brain state aberrations in each patient [30,31].

By employing advanced machine learning algorithms to analyze fMRI data, scientists have begun to map out these key control points within the brain’s network architecture [32]. This mapping not only highlights the regions implicated in MDD but also unveils potential targets for novel therapeutic interventions designed to enhance or restore the brain’s controllability [17,33]. Notably, the identification of such pivotal brain regions opens the door to precision medicine approaches in psychiatry. This helps in developing an individualized intervention plan for each person’s different neurological dysfunctions, as opposed to the traditional uniform treatment strategy. For instance, techniques such as transcranial magnetic stimulation (TMS) or deep brain stimulation (DBS) could be refined to selectively modulate the activity of these key control points, thereby optimizing the therapeutic outcomes for patients with MDD [34,35]. This burgeoning field of research, which merges network science, neuroimaging, and machine learning, not only enhances our comprehension of MDD’s complex neurobiological underpinnings but also charts a course toward more effective and personalized treatment strategies. As we continue to unravel the intricacies of brain network controllability and its implications for MDD, we edge closer to realizing the full potential of precision psychiatry in transforming the lives of those affected by this debilitating disorder.

This paper aims to advance the understanding of MDD by leveraging fMRI data to analyze brain network connectivity and controllability. We hypothesize that disruptions in specific brain networks play a crucial role in the pathophysiology of MDD and that these networks’ controllability properties could illuminate pathways for novel therapeutic interventions. Our study builds on these methodologies; we will explore the alterations in brain network connectivity associated with MDD and examine the network’s controllability characteristics. By combining detailed fMRI data analysis with advanced machine learning techniques, we seek to map out critical control points within the brain’s networks, offering new insights into the brain bases of MDD and enhancing the feasibility of precision medicine approaches in psychiatry.

## 2. Methods

### 2.1. Participants

This study was conducted with a total of 286 participants, divided into two groups: the healthy control group (HC) consisting of 130 individuals, and the Major Depressive Disorder (MDD) group comprising 156 individuals. The age range for both groups was 18 to 30 years. The HC group included 73 females and 57 males, while the MDD group had 85 females and 71 males.

Participants were recruited from the Early Intervention Unit of the Affiliated Nanjing Brain Hospital of Nanjing Medical University in China. Exclusion criteria for all participants included a history of neurological illness, significant head trauma, substance abuse, or any contraindications to magnetic resonance imaging (MRI), such as pacemakers or claustrophobia. Additionally, participants with any psychiatric comorbidity other than MDD were excluded from this study.

The inclusion criteria for the HC group stipulated that participants must have no history of psychiatric illness, as confirmed by the Structured Clinical Interview for DSM-IV (SCID) [36], and no first-degree relatives with a history of psychiatric disorders. The inclusion criteria for the MDD group stipulated that participants must have a current diagnosis of MDD, as determined by the SCID, and moderate to severe depressive symptoms, as indicated by a score of 14 or higher on the Hamilton Depression Rating Scale (HAMD) [37]. All participants signed a written informed consent form, and this study was approved by the Ethics Review Committee of Nanjing Medical University.

### 2.2. MRI Data Acquisition

The MRI scans were performed in the Department of Radiology, Nanjing Brain Hospital, Nanjing Medical University, and were acquired using a MAGNETOM Prisma 3.0T superconducting magnetic resonance imaging scanner manufactured by Siemens AG, Germany, and operated by professional radiologists. The equipment utilized EPI hardware and software, along with a 64-channel neurofunctional coil. The software platform used was syngo.via. All subjects underwent resting-state MRI. The parameters for resting-state functional imaging included TR = 500 ms, TE = 30 ms, FOV = 224 × 224 mm^2^, voxel size = 3.5 × 3.5 × 3.5 mm^3^, and interlayer spacing = 0 mm. A total of 35 layers were scanned, accumulating 960 layers, with the total scan duration being 8 min. The parameters for high-resolution structural imaging were TR = 2530 ms, TE = 2.98 ms, field of view (FOV) = 256 × 224 mm², voxel dimensions = 0.5 × 0.5 × 1 mm³, flip angle = 7°, and slice thickness = 1 mm, scanning 192 layers in 6 min. For diffusion tensor imaging (DTI), the settings were TR = 2800 ms, TE = 63 ms, FOV = 216 × 216 mm², voxel size = 2 × 2 × 2 mm³, and slice thickness = 2 mm, with no layer spacing, applying gradients in 64 directions and a b-value of 1000 s/mm², covering 75 layers in 3 min and 28 s.

### 2.3. Data Processing

The resting-state functional MRI data preprocessing procedure was performed using the Statistical Parametric Mapping 12 (SPM12, http://www.fil.ion.ucl.ac.uk/spm, accessed on 10 May 2022) [38] and Data Processing Assistant for R-fMRI (DPARSF; http://www.restfmri.net/forum/DPARSF, accessed on 10 May 2022) toolkits [39]. The main steps were as follows: (1) the raw DICOM format data were converted to Nifti format; (2) the effects of machine startup and subject acclimatization processes on the results were excluded by removing the first 10 time points and retaining the resting-state sequence data at 950 time points; (3) the remaining images were corrected for slice temporal aberration; and (4) head-motion correction was performed with > 3 mm displacement and/or 3° rotations were excluded. The images were spatially normalized by converting them to standard EPI templates in the Montreal Neurological Institute (MNI) space with a voxel size of 3 × 3 × 3 mm. Spatial smoothing was applied using a Full Width at Half Maximum (FWHM) of 6 mm. To mitigate the effects of low-frequency drift and high-frequency physiological noise, the images were linearly regressed and temporally band-pass filtered between 0.01 and 0.08 Hz. The ROI was defined based on the automated anatomical labeling (AAL) template [40] included in the DPABI toolkit, which had been resampled to a voxel size of 3 × 3 × 3 mm^3^. Functional connectivity was computed through a correlation analysis between ROIs powered by DPABI software (v3.1, http://rfmri.org/dpabi, accessed on 10 May 2022).

### 2.4. Average Controllability Analysis

The concept of average controllability quantifies the ability of a network’s nodal dynamics to be steered into diverse states using minimal input energy [18]. In the context of brain functional connectivity, it serves as a measure of how easily the brain’s activity pattern can transition between different functional states, which is crucial for understanding brain flexibility and resilience in health and disease.

To translate functional connectivity matrices into controllability metrics, we utilized the framework of network control theory [19]. Specifically, we modeled the brain as a linear time-invariant system [27],

$$\frac{dx(t)}{dt}=Ax\left(t\right)+Bu(t)$$

where the state of the system at any given time is represented by the pattern of neural activity across the network. $x\left(t\right)={\left(x_{1}\left(t\right),\ldots ,x_{N}\left(t\right)\right)}^{T}$ denotes the state of all nodes at time t, A is the transposed adjacency matrix of the system, $u\left(t\right)={\left(u_{1}\left(t\right),\ldots ,u_{M}\left(t\right)\right)}^{T}$ is the set of external control signals, and B is the input matrix specifying where control signals are applied onto the network.

The average controllability of each node (ROI) in the network was then computed as the inverse of the average input energy required to transition the system from its initial state to a target state over one time step [41]. This computation was performed using the control energy formula, which integrates the network’s adjacency matrix and the controllability Gramian $W_{\kappa }$, where

$$W_{\kappa }=\sum_{t=0}^{\infty }A^{\tau }B_{\kappa }B_{\kappa }^{T}A^{\tau }$$

The analysis was coded in Python, and the average controllability metric was calculated by the nctpy package [42]. For each participant, the average controllability was calculated for every ROI in the brain, yielding a controllability profile that captured the potential of each region to influence whole-brain dynamics. Statistical comparisons of average controllability between the HC and MDD groups were performed using two-sample *t*-tests. Regions showing significant differences in average controllability between groups were further analyzed to elucidate their roles in the pathophysiology of MDD and their potential as biomarkers for the disorder.

### 2.5. Predictive Model

To predict MDD presence based on brain controllability metrics, we employed a fully connected artificial neural network (ANN) model [43]. The ANN architecture was designed to capture the complex, non-linear relationships between brain connectivity features and the clinical status of MDD. Our ANN consisted of an input layer, six hidden layers, and an output layer. The input layer received a vector of features derived from the average controllability metric for each participant.

The model incorporated dropout layers to prevent overfitting [44], with a dropout rate of 0.25. Each hidden layer utilized a Rectified Linear Unit (ReLU) activation function to introduce non-linearity into the model, facilitating the learning of complex patterns in the data:

$$f\left(z\right)=\max(0,z)$$

where z is the input to a neuron within the network. The output layer used a sigmoid activation function [45] to generate a probability score P(y=1|x) indicating the likelihood of MDD presence:

$$P\left(y=1|x\right)=\frac{1}{1+e^{-w^{T}x-b}}$$

where w represents the weight vector, x is the input feature vector, and b is the bias.

Training of the ANN was performed using ten-fold cross-validation [46] to ensure the model’s generalizability across unseen data. In each fold, the data were split into training and testing sets, with the model being trained on the training set and evaluated on the testing set. This process was repeated ten times, with each fold serving as the testing set once. The model’s performance was assessed using accuracy, sensitivity, specificity, and the area under the receiver operating characteristic (ROC) curve as metrics, where the ROC is defined by plotting the true positive rate (*TPR*) against the false positive rate (*FPR*) at various threshold settings:

$$TPR=\frac{TP}{TP+FN},\;FPR=\frac{FP}{FP+TN}$$

where TP, TN, FP, and FN represent the numbers of true positives, true negatives, false positives, and false negatives, respectively.

This ANN approach allowed us to harness the predictive power of complex brain connectivity data in identifying individuals with MDD, offering insights into potential biomarkers and contributing to the development of diagnostic tools in clinical settings.

## 3. Results

### 3.1. Demographics and Clinical Characteristics

This study involved a total of 286 participants, divided into an HC group of 130 individuals and an MDD group consisting of 156 individuals, with an age range of 18 to 28 years for both groups. Both groups were matched for age and gender, with no significant differences observed in the mean age of the HC (mean ± SD: 24.2 ± 2.36) and MDD (23.7 ± 2.59) groups (*p* = 0.52). There was also no significant difference in gender distribution between the two groups (*p* = 0.46).

Clinical characteristics, as assessed by the HAMD, revealed a significant difference in scores between the MDD group (mean ± SD: 22.28 ± 6.39) and the HC group, which was expectedly lower (1.29 ± 1.94); *p* < 0.001. This difference underscores the clinical distinction between the groups and validates the inclusion criteria for the MDD group based on the severity of depressive symptoms. Additionally, the sex-specific analysis of HAMD scores revealed that females in the MDD group had slightly higher scores on average (mean ± SD: 23.03 ± 5.64) compared to males (mean ± SD: 21.60 ± 6.44); however, there was no significant difference between the two genders (*p* = 0.14). These results underscore the appropriateness of the composition and gender distribution of our participants for this study.

### 3.2. Brain Average Controllability in HC and MDD

**Figure 1:**
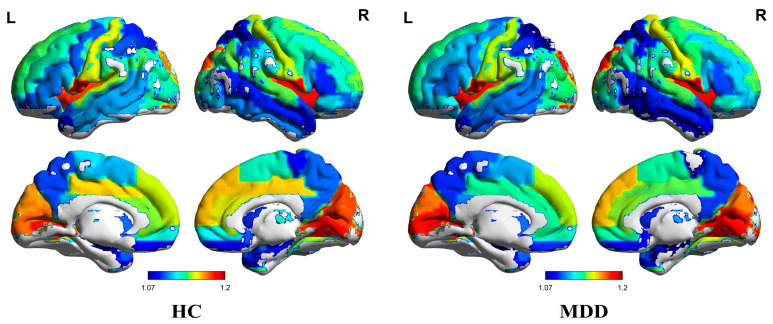
Average controllability means of the HC group (**left**) and MDD group (**right**). The color of the brain area represents the value of average controllability. The labels “L” and “R” in the figure caption indicate the left and right hemispheres of the brain, respectively.

In our study, we computed the average controllability for each brain region of every individual within the HC and MDD cohorts. The mean average controllability of each region across participants within a group was then calculated and visually represented in Figure 1 and the Supplementary Materials. It became evident that the overarching patterns of average controllability showed no significant divergence between groups, which may imply the retention of global brain dynamics irrespective of the disorder. Nevertheless, a more nuanced analysis brought to light localized variations that warrant attention.

Specifically, we calculated the average controllability difference between the MDD group and the HC group in the corresponding brain regions (by average controllability value in HC minus average controllability in MDD). We observed in the MDD group, compared to HC, the top five elevations in the average controllability in regions such as the *Frontal_Mid_Orb_L* (difference: HC-MDD = −0.0165), *SupraMarginal_L* (difference: HC-MDD = −0.0161), *Occipital_Sup_L* (difference: HC-MDD = −0.0148), *Frontal_Inf_Orb_L* (difference: HC-MDD = −0.0148) and *Calcarine_L* (difference: HC-MDD = −0.0122), while the top five decreases were noted in areas like the *Temporal_Inf_R* (difference: HC-MDD = 0.0284), *Cingulum_Mid_L* (difference: HC-MDD = 0.0243), *Thalamus_R* (difference: HC-MDD = 0.0224), *Parietal_Sup_L* (difference: HC-MDD = 0.0205) and *Thalamus_L* (difference: HC-MDD = 0.0184). The enhancement in the average controllability might hint at compensatory mechanisms or reflect the underlying pathophysiological shifts associated with MDD. In contrast, the diminished controllability could indicate a reduced capacity of these regions to facilitate transitions between different brain states, potentially contributing to the clinical manifestations of MDD.

**Figure 2:**
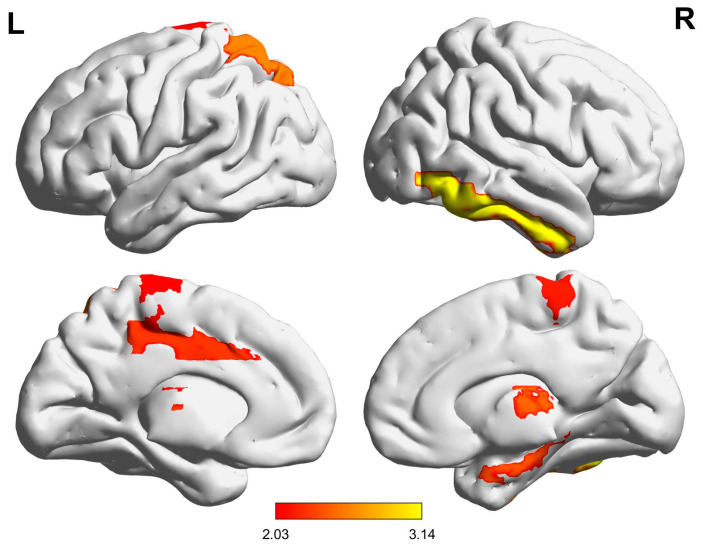
Average controllability differences in brain areas between the HC group and MDD group (*p* < 0.05). The color of the brain area indicates the t value. The labels “L” and “R” in the figure caption indicate the left and right hemispheres of the brain, respectively.

**Figure 3:**
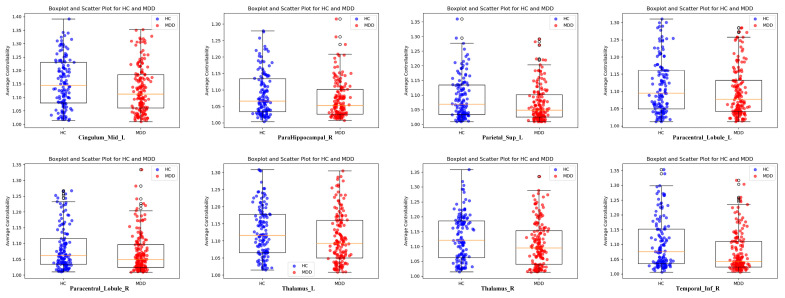
Scatter distribution diagram of average controllability in different brain areas between the HC group and the MDD group. The orange line represents the median of the dataset for each group.

To further probe into the regional differences between the HC and MDD groups, we conducted a statistical analysis. Utilizing a two-sample t-test to compare the mean average controllability of each brain area between the groups, we identified eight regions where the differences were statistically significant (*p* < 0.05), including *Cingulum_Mid_L*, *ParaHippocampal_R*, *Parietal_Sup_L*, *Paracentral_Lobule_L*, *Paracentral_Lobule_R*, *Thalamus_L*, *Thalamus_R,* and *Temporal_Inf_R*, as delineated in the Supplementary Materials and Table 1 and illustrated in Figure 2. The average controllability scatter plot of these eight brain regions is shown in Figure 3. In addition to the *t*-test, we also employed the Mann–Whitney U test to analyze differences across brain regions. The result shows that, beyond the initial eight brain regions, four additional regions—*Hippocampus_R*, *Frontal_Mid_R*, *ParaHippocampal_L*, and *Cingulum_Mid_R*—demonstrated differences between the MDD group and the HC group. However, after applying the FDR correction for multiple comparisons, only *Temporal_Inf_R* remained significantly different, with a value of 0.036. Despite these limitations, the differential average controllability observed across these eight brain regions under both statistical tests may encapsulate the underlying mechanistic distinctions between MDD and HC, serving as potential biomarkers or signature regions for differentiating between the two cohorts.

### 3.3. Predictive Analysis of Average Controllability in Difference Brain Regions as Biomarkers

**Figure 4:**
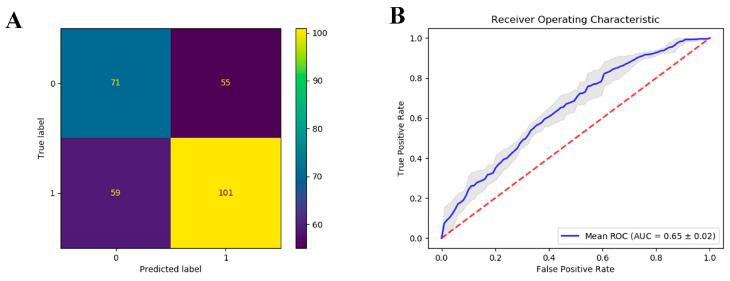
Confusion matrix and ROC curve results of the prediction model. (**A**) Confusion matrix; (**B**) ROC curve results. Red dashed line represents the diagonal line of no-discrimination. It serves as a baseline to measure how well the predictive model performs compared to random results. Grey shaded area around the blue ROC curve represents the confidence interval of the ROC curve estimates based on cross-validation.

In our analysis, the average controllability metrics from brain regions with significant differences between the HC and MDD groups served as input features for our predictive model. Figure 4A illustrates the prediction outcomes of this model for our cohort, where 0 denotes the HC group and 1 represents the MDD group. The horizontal axis signifies the predicted classification, while the vertical axis corresponds to the actual diagnostic label of the subjects. The result reveals that the correct classification rate for the MDD group is 0.63, suggesting a low false positive rate. The overall accuracy of the model stands at 0.60, highlighting its capacity to distinguish between HC and MDD individuals with substantial reliability.

Furthermore, the model’s performance across each fold of a rigorous 10-fold cross-validation is documented in Figure 4B. The ROC curve, illustrated for each fold, consistently lies above the threshold of 0.5, thereby confirming the efficacy of the classifier’s training for all subsets of the data. These results collectively underscore the potential of average controllability measures in brain regions as reliable biomarkers for detecting MDD.

## 4. Discussion

Our research aimed to identify neuroimaging biomarkers for MDD by employing network controllability and machine learning techniques to analyze fMRI data, leading to the identification of eight key brain regions with significantly altered controllability. These regions, including the limbic system, thalamus, superior parietal lobule, precuneus, cingulate cortex, and temporal poles, suggest a distinct pattern of brain dysregulation in individuals with MDD. The patterns constituted by the average controllability in these regions differ from those in healthy controls. Based on our findings, we hypothesize that alterations in network controllability within certain brain regions, such as the anterior cingulate cortex, thalamus, and limbic system, significantly contribute to the pathophysiology of MDD. These regions, identified as having altered average controllability, are hypothesized to play a pivotal role in mood regulation and cognitive processing dysfunctions observed in MDD. The specific changes in the ability of these regions to influence neural state transitions may disrupt the brain’s capacity to maintain cognitive and emotional equilibrium, thus leading to the symptoms characteristic of MDD.

In our study, we pinpoint several key regions implicated in the complex neurobiology of MDD, demonstrating their critical roles in emotional regulation, cognitive processing, and sensory integration. These regions include the anterior cingulate gyrus (ACC), parahippocampal gyrus, superior parietal lobe, paracentral lobule, inferior temporal gyrus, and thalamus. Notably, the ACC is integral to cognitive and emotional functions, influencing affective symptoms and playing a crucial role in depression’s pathophysiology and treatment mechanisms [47,48]. The parahippocampal gyrus, essential for memory and emotional regulation, also shows significant structural differences in schizophrenia compared to controls, underscoring its broader neuropsychiatric relevance [49,50]. Furthermore, the superior parietal lobe and paracentral lobule, components of the somatosensory cortex, are involved in the multistep process of emotion generation and regulation [51,52]. The temporal lobe, part of the default mode network, is crucial for various cognitive functions and has been linked to treatment outcomes in mood disorders [53,54]. The thalamus, central to mood regulation circuitry, might also play a significant role in the pathophysiological processes of mood and psychotic disorders [55,56,57,58]. Enhanced connectivity involving the inferior temporal gyrus and dorsolateral prefrontal cortex correlates with mood disorder symptoms [59]. Collectively, these regions highlight a network of neural activities that contribute to the typical symptomatology of MDD, underscoring the necessity of a systems-level approach to understanding and treating this complex disorder.

The concept of network controllability, central to our study, offers a significant leap forward in understanding the complex dynamics of brain networks, especially in the context of disorders such as MDD. The ability to modulate specific regions within the brain network not only provides insights into the neurobiological basis of MDD but also opens avenues for innovative therapeutic strategies. By identifying and targeting these control points, interventions can be designed to alter the brain’s path from a diseased state towards normal functioning, thus offering hope for more effective treatments.

Despite the robust methodology used, our study’s limitations include the static nature of fMRI data, which constrains our ability to capture dynamic changes over time. Additionally, our sample was restricted to a specific demographic, which could restrict the applicability of the findings to a wider population. Future investigations should aim to replicate these findings in longitudinally collected data to understand how these control changes evolve with disease progression or treatment. The sample size should also be increased to obtain more significant statistical significance. Moreover, using only fMRI data as a biomarker to distinguish between MDD and HC may have certain limitations. For instance, Lai et al. reported that a single neuroimaging index is an unreliable predictor of MDD treatment response [60], whereas Kennis et al. found that cortisol is a more accurate predictor of depressive episodes and relapses than neuroimaging markers alone [61]. Therefore, our subsequent research will integrate genetics, epigenetics, and longitudinal clinical data. Employing multi-modal data will also enhance our understanding of the causal pathways in MDD. Finally, the use of drugs will also have an impact on the research results [62,63]. In subsequent studies, drug factors should be excluded as much as possible to ensure the quality of the research.

## 5. Conclusions

This study underscores the utility of machine learning and network control theory in identifying potential neuroimaging biomarkers for MDD. By delineating specific brain regions with altered controllability, we not only enhance the understanding of the neurobiological underpinnings of MDD but also pave the way for developing targeted therapeutic strategies based on these biomarkers.

## Figures and Tables

**Table 1:** Average controllability of 8 brain areas as biomarkers. *Ave_control_HC* is the mean value of average controllability in the HC group. *Ave_control_MDD* is the mean value of average controllability in the MDD group. *HC-MDD* is the difference between the two.

ID	Name	*Ave_control_HC*	*Ave_control_MDD*	*HC-MDD*	*p* Value	*T* Value
33	Cingulum_Mid_L	1.155	1.131	0.024	0.0235	2.2778
40	ParaHippocampal_R	1.088	1.070	0.017	0.0161	2.4204
59	Parietal_Sup_L	1.091	1.070	0.020	0.0092	2.6217
69	Paracentral_Lobule_L	1.115	1.098	0.018	0.0428	2.0349
70	Paracentral_Lobule_R	1.087	1.070	0.017	0.0302	2.1778
77	Thalamus_L	1.126	1.108	0.018	0.0342	2.1276
78	Thalamus_R	1.130	1.108	0.022	0.014	2.4729
90	Temporal_Inf_R	1.103	1.075	0.028	0.0019	3.1404

## Data Availability

The dataset supporting the conclusions of this article will be made available by the authors upon reasonable request. The data are not publicly available due to ongoing analysis and additional research projects that are building upon this dataset.

## Supplementary Materials

The following supporting information can be downloaded at: https://www.mdpi.com/article/10.3390/brainsci14050509/s1, Table S1: Average controllability results across all brain regions.
